# Resection of recurrent hepatocellular carcinoma with thrombi in the inferior vena cava, right atrium, and phrenic vein: a report of three cases

**DOI:** 10.1186/s12957-020-01914-8

**Published:** 2020-06-22

**Authors:** Koichi Tomita, Motohide Shimazu, Kiminori Takano, Takahiro Gunji, Yosuke Ozawa, Toru Sano, Naokazu Chiba, Yuta Abe, Shigeyuki Kawachi

**Affiliations:** 1 Department of Digestive and Transplantation Surgery, Tokyo Medical University Hachioji Medical Center, 1163 Tatemachi, Hachioji-shi, Tokyo, 193-0998 Japan; 2 Department of Surgery, Tama Kyuryo Hospital, 1491 Shimooyamada, Machida-shi, Tokyo, 194-0297 Japan; 3 Department of Surgery, Hiratsuka City Hospital, 1-19-1 Minamihara, Hiratsuka-shi, Kanagawa 254-0065 Japan; 4 Department of Surgery, Keio University School of Medicine, 35 Shinanomachi, Shinjuku-ku, Tokyo, 160-8582 Japan

**Keywords:** Hepatocellular carcinoma, Inferior vena cava, Phrenic vein, Right atrium, Tumor thrombus

## Abstract

**Background:**

Prognosis for patients with advanced hepatocellular carcinoma with a tumor thrombus in the inferior vena cava or right atrium is extremely poor due to cancer progression, pulmonary embolism, and congestion of the circulatory system caused by right heart failure. Surgical resection of the tumor thrombi may potentially yield better results than non-surgical treatments through prevention of sudden death. However, the benefits of surgical resection in patients with hepatocellular carcinoma and a tumor thrombus extending to the inferior vena cava, right atrium, and potentially in the phrenic vein are unclear. Here, we report three such cases.

**Case presentation:**

Of the total 136 patients who underwent hepatectomies for hepatocellular carcinoma in our institution, three patients with prior hepatectomies and recurrent hepatocellular carcinoma had tumor thrombi in the inferior vena cava, right atrium, and phrenic vein. Surgical resections were performed, as there was a possibility of sudden death, despite the risk of leaving residual tumor. For all patients, we performed resection of the tumor thrombi in the inferior vena cava and right atrium and combined diaphragm resection. Surgical resection was performed using the total hepatic vascular exclusion technique in all cases. Additional passive veno-venous bypass was also performed in two cases, in which complete tumor resections could not be achieved. The microscopic surgical margins of the combined resected diaphragms were positive in all cases. Progression-free survival was 20.2, 3.8, and 9.5 months for case 1, 2, and 3, respectively. The respective overall postoperative survival was 98.0, 38.9, and 30.9 months. The patients died due to liver cirrhosis, acute heart failure, and hepatocellular carcinoma, respectively. Sudden death did not occur for any of the patients.

**Conclusion:**

Surgical resections may extend prognosis for patients with recurrent hepatocellular carcinoma with tumor thrombi in the inferior vena cava, right atrium, and phrenic vein, although the indications should be considered carefully.

## Background

The prognosis for patients with advanced hepatocellular carcinoma (HCC) with a tumor thrombus in the inferior vena cava (IVC) or right atrium (RA), defined as Vv3 by the American Joint Committee on Cancer and the Union for International Cancer Control [1], is extremely poor due to cancer progression, pulmonary embolism-related sudden death [2], and congestion of the circulatory system caused by right heart failure [3].

According to the European Association for the Study of the Liver guidelines, Vv3 cases are classified as Barcelona-Clinic Liver Cancer Stage C, and their recommended treatment is “systemic therapy” [4]. The American Association for the Study of Liver Disease also suggests the same treatment [5]. Conversely, surgical resections of the tumor thrombi may potentially yield better results than non-surgical treatments through prevention of sudden death, with some patients achieving long-term survival [6].

A tumor thrombus may appear not only in the IVC and RA, but also in the phrenic vein. The effect of surgical resections in these cases is unclear. Herein, we describe three patients with recurrent HCC and a tumor thrombus in the IVC, RA, and phrenic vein who underwent surgical resection of the tumor thrombus combined with diaphragm resection. We aimed to determine whether performing surgical resections for this tumor type would improve patient prognosis.

## Case presentations

### Patients

A total of 136 patients underwent hepatectomies for HCC at our center between November 2006 and December 2019. Of these patients, tumors extending to the IVC were diagnosed in four. We performed surgical resections for these patients, as there was a possibility of sudden death due to pulmonary embolisms or cardiac failure caused by the tumor thrombus, although the possibility of leaving residual tumor remained.

Three of the four patients had recurrent HCC and are presented below. According to dynamic-enhanced computed tomography imaging or magnetic resonance imaging, regions of low density or low intensity were recognized as tumor thrombi in the IVC, RA, and phrenic vein. The patient features are listed in Table 1.

**Table 1:** Course of the three patients with tumor thrombi in the IVC, RA, and phrenic vein

Case no.		1	2	3
Age, years		69	40	75
Sex		Male	Female	Female
Preoperative information	Etiology	Alcoholic	Unknown	HCV
	Past treatment	1. TACE (left lobe, S8) 2. Left hepatectomy 3. RFA (S8)	1. Lateral sectionectomy 2. TACE (S7, S8)	1. TACE 2. PEIT, RFA 3. TACE, RFA (S7, 8)
	Child-Pugh (score, grade)	7, B	5, A	6, A
	Tumor markers before surgery	AFP 912 ng/mL PIVKA-II 294 U/mL	AFP 15 ng/mL PIVKA-II 25681 U/mL	AFP 9521 ng/mL PIVKA-II 4476 U/mL
	Tumor location	IVC, RA, diaphragm	S7, IVC, RA, diaphragm	S7, IVC, RA, diaphragm
Operative findings	Surgical procedure	RA thrombectomy Diaphragm resection	RA thrombectomy Diaphragm resection Posterior sectionectomy	RA thrombectomy Diaphragm resection
	Hepatectomy	−	+	−
	Operation time	9 h 2 min	10 h 0 min	8 h 15 min
	THVE time, min	26	21	25
	V-V bypass	−	+	+
	Bleeding, *g*	2705	1080	4230
Pathological findings	Differentiation	Poor	Moderate	Poor
	Surgical margin	+, diaphragm	+, diaphragm	+, diaphragm
Postoperative course	Complication	Pleural effusion	Pleural effusion	Respiratory failure Pleural effusion
	Postoperative stay, days	42	14	43
	Tumor marker (3POM)	AFP 89 ng/mL PIVKA-II 79 U/mL	AFP 16 ng/mL PIVKA-II 633 U/mL	AFP 573 ng/mL PIVKA-II 3021 U/mL
	CTx after surgery	Sorafenib	Sorafenib	Sorafenib
	Recurrence	Parasternal site	Lung, bone	Remnant liver (S7)
	PFS, months	20.2	3.8	9.5
	Death	Liver cirrhosis	Acute heart failure	HCC
	Postoperative survival, months	98.0	38.9	30.9

*Abbreviations: IVC* inferior vena cava, *RA* right atrium, *HCV* hepatitis C virus, *TACE* trans-catheter arterial chemoembolization, *PEIT* percutaneous ethanol injection therapy, *RFA* radiofrequency ablation, *AFP* alpha-fetoprotein, *PIVKA-II* protein-induced by vitamin K absence-II, *THVE* total hepatic vascular exclusion, *POM* post-operative month, *CTx* chemotherapy, *PFS* progression free survival, *HCC* hepatocellular carcinoma

This study was approved by the institutional review board of Tokyo Medical University (T2019-0256). Written informed consent was obtained from all patients.

### Case 1

**Fig. 1:**
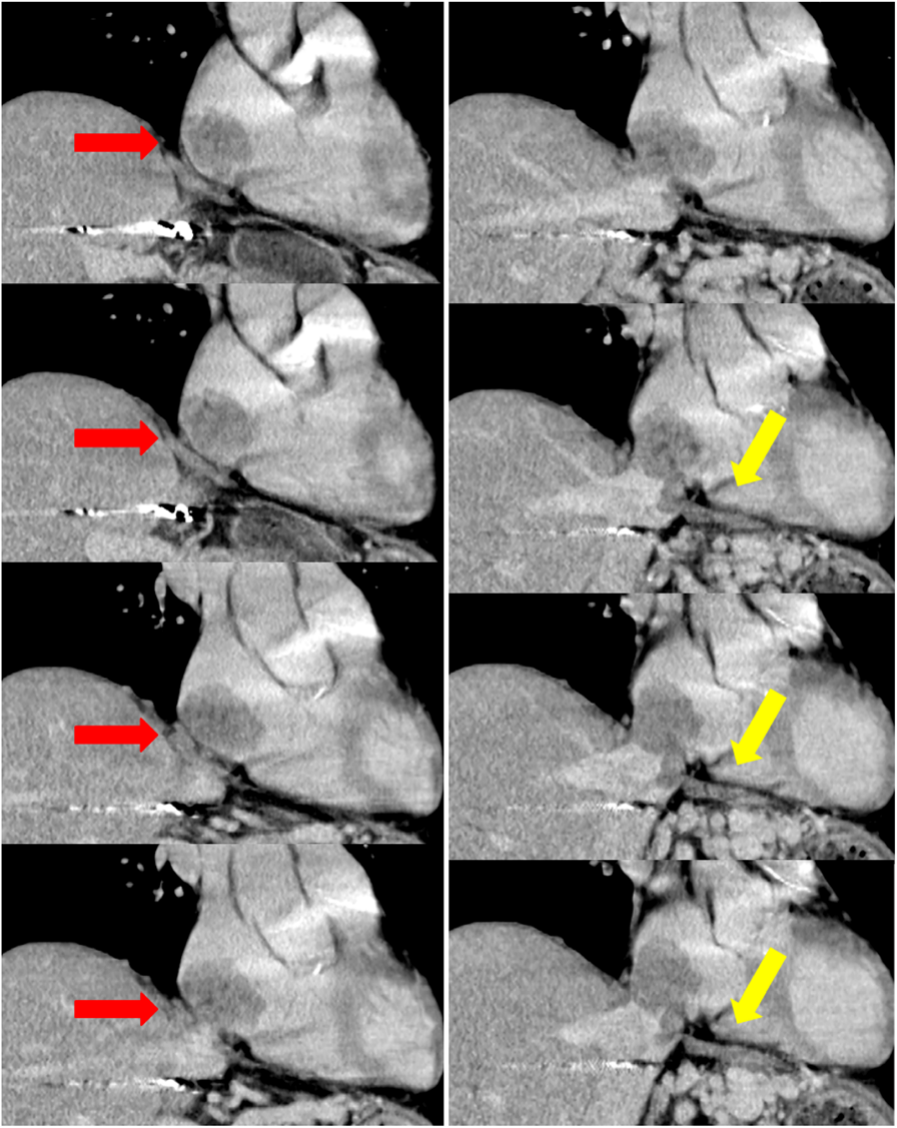
Dynamic-enhanced computed tomography imaging of case 1 (reconstruction for the coronal axis). A tumor thrombus was detected in both the inferior vena cava (IVC) and right atrium (RA) (red arrow), with extension to the left phrenic vein (yellow arrow)

The patient was a 69-year-old man with a history of heavy alcohol use. Transarterial chemoembolization (TACE) had been performed twice for HCC at another hospital, 7 years prior to our surgery. Additionally, a left hepatectomy was performed 1 year before our surgery. After the hepatectomy, recurrent HCC was recognized in segment (S) 8, and radiofrequency ablation was performed. Subsequently, a tumor thrombus in the IVC, RA, and phrenic vein was detected (Fig. 1). The thrombus was close to the stump of the remnant liver and left hepatic vein, though not detectable inside the remnant liver.

### Case 2

**Fig. 2:**
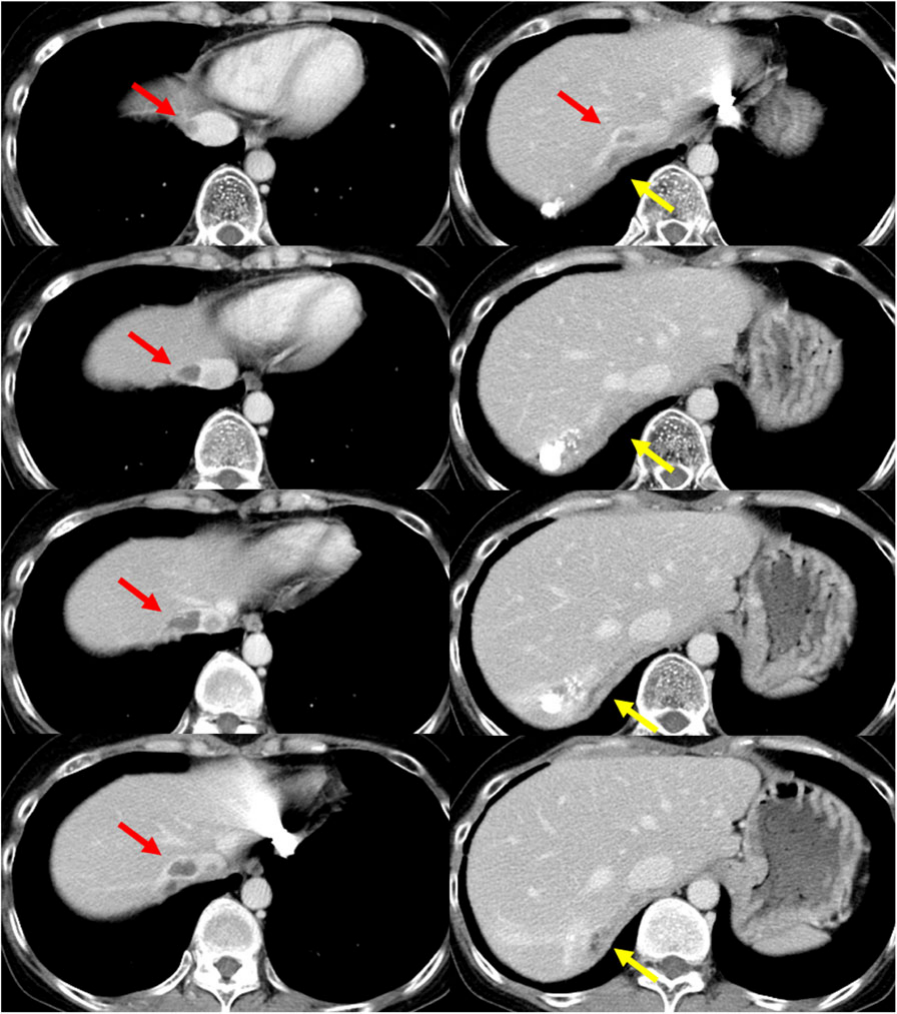
Dynamic-enhanced computed tomography imaging of case 2. A tumor thrombus was detected in the right hepatic vein, inferior vena cava (IVC), and right atrium (RA; red arrow), as well as in the right phrenic vein (yellow arrow)

The patient was a 40-year-old woman with HCC of unknown etiology. Lateral sectionectomy had been performed for HCC 11 years before our surgery at another hospital. Afterwards, TACE was repeatedly performed for HCC of S7 and S8. However, a tumor thrombus was recognized near the S7 tumor (Fig. 2).

### Case 3

**Fig. 3:**
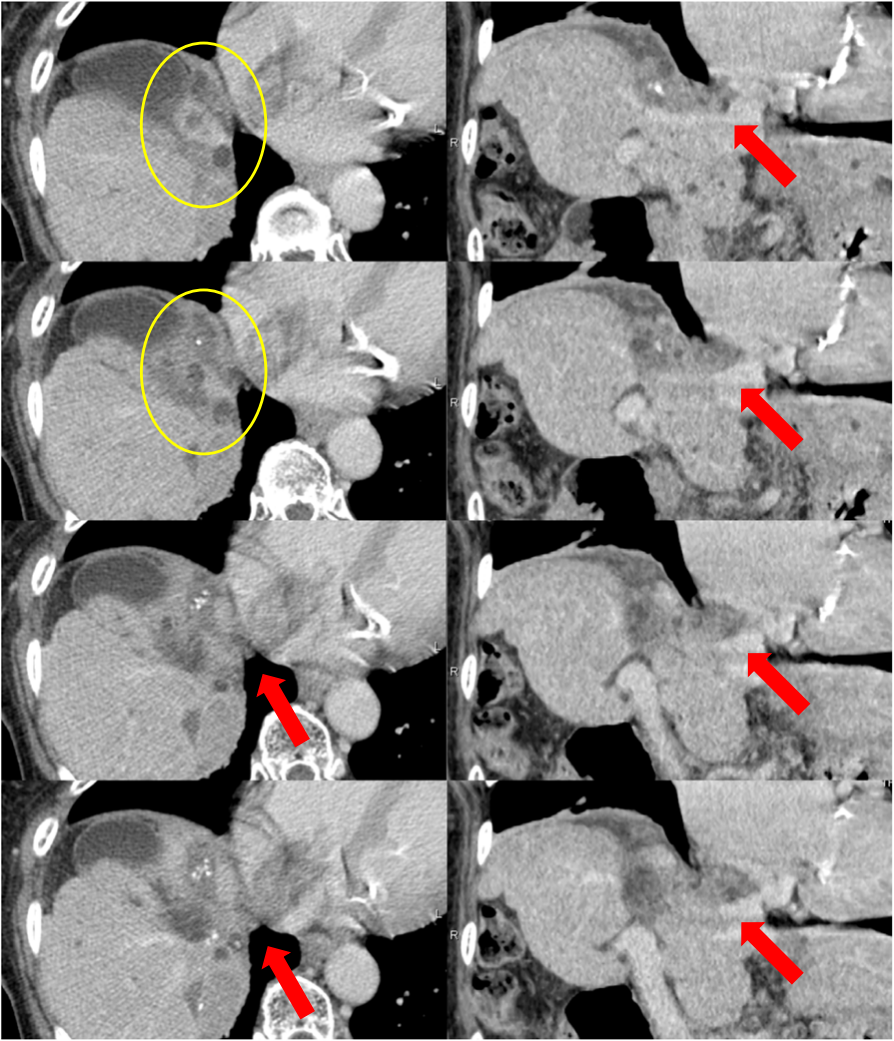
Dynamic-enhanced computed tomography imaging of case 3. Tumor thrombus was detected in the right hepatic vein, inferior vena cava (IVC), and right atrium (RA; red arrow), as well as in the right phrenic vein (yellow circle). The left column shows the axial view, and the right column shows the coronal view

The patient was a 75-year-old woman with chronic hepatitis C viral infection. Repeated TACE, percutaneous ethanol injection therapy, and radiofrequency ablation had been performed for multiple HCCs of both lobes during 8 years before our surgery. After these treatments, a low-density area was recognized under the diaphragm with a tumor thrombus (Fig. 3).

### Surgical procedure

After obtaining informed consent, surgical resection was selected for these three patients for the prevention of sudden death due to tumor thrombosis. Although there was a chance of complete tumor resection, this outcome was expected to be difficult to achieve. Therefore, the possibility of the surgery being non-curative was fully explained to the patients before surgery.

Surgical resection of the tumor thrombus extending from the IVC to the RA was performed using the total hepatic vascular exclusion (THVE) technique without cardiopulmonary bypass. The vasculature around the liver, including the hepatoduodenal ligament, infrahepatic IVC, and suprahepatic IVC, were encircled and excluded during the reconstruction of the IVC. Further, draining of the left adrenal and lumbar veins to the IVC was divided when possible. Intraoperatively, the RA was clamped instead of the suprahepatic IVC. Intra-abdominal and transesophageal ultrasound sonography were used during surgery to avoid damaging the tumor thrombus and tricuspid valve. RA clamping was performed beneath the right coronary artery and coronary sinus.

In cases 2 and 3, the blood pressure was unstable during THVE due to clamping of the IVC, and an additional passive veno-venous bypass with a heparinized catheter was used. The portal flow was passively shunted from the inferior mesenteric to the left axial vein to prevent intestinal congestion. The IVC flow was also extracted from the right femoral vein and passively directed to the left axial vein with portal flow by a Y-shaped connector. During the veno-venous bypass, systemic heparinization or the use of a Bio-Pump was not necessary.

**Fig. 4:**
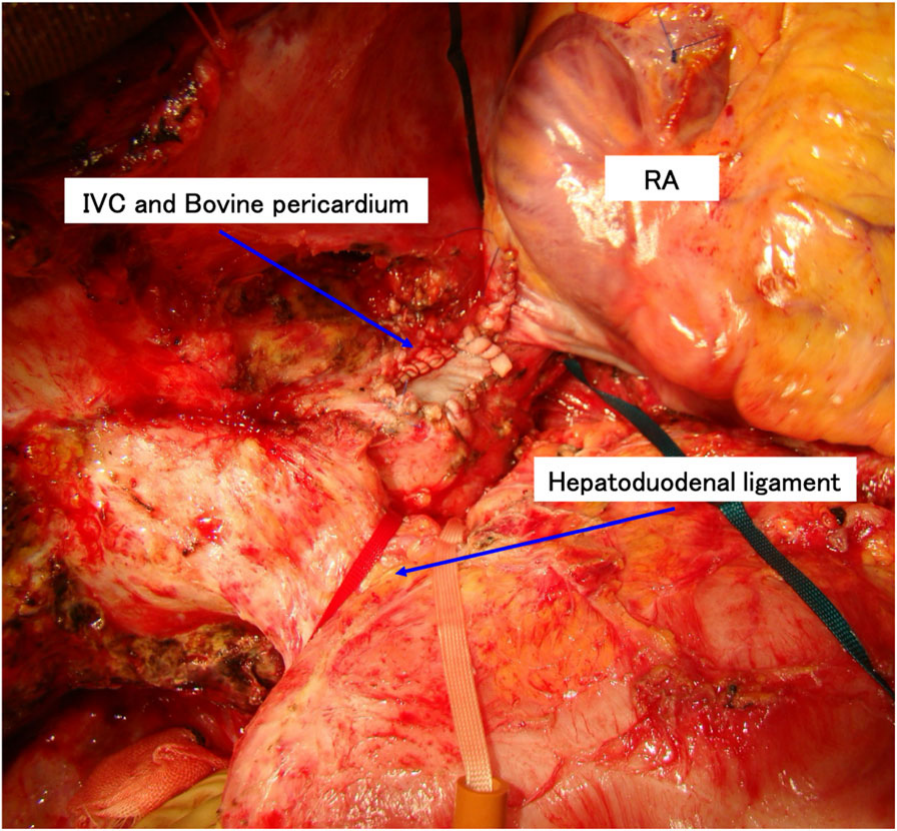
Surgical view of case 1. View after resection of the tumor thrombus and reconstruction of the inferior vena cava (IVC) and right atrium (RA) wall defect, using bovine pericardium

Bovine pericardium (Edwards Lifesciences®, Irvine, CA, USA) was used during the reconstruction of the RA and IVC wall defect (Fig. 4); when needed, it was also used for reconstruction of diaphragm defects.

### Operative findings

In all three patients, the tumor thrombi of the IVA and RA were considered to have extended to the phrenic vein. Thrombectomies in the IVC and RA and combined diaphragm resections were performed. For case 2, a hepatic posterior sectionectomy was also performed for resection of the intrahepatic HCC. For case 1, macroscopic complete resection of the inferior phrenic vein with tumor thrombus was performed, although the microscopic surgical margin was proven to be positive. For case 2, the microscopic tumors extended broadly inside the diaphragm and could not be completely resected, although the diaphragm was resected to the extent possible. For case 3, the intrahepatic tumor mass was not capsulated, and a part of it could not be resected because it was close to the umbilical portion.

### Pathological findings

**Fig. 5:**
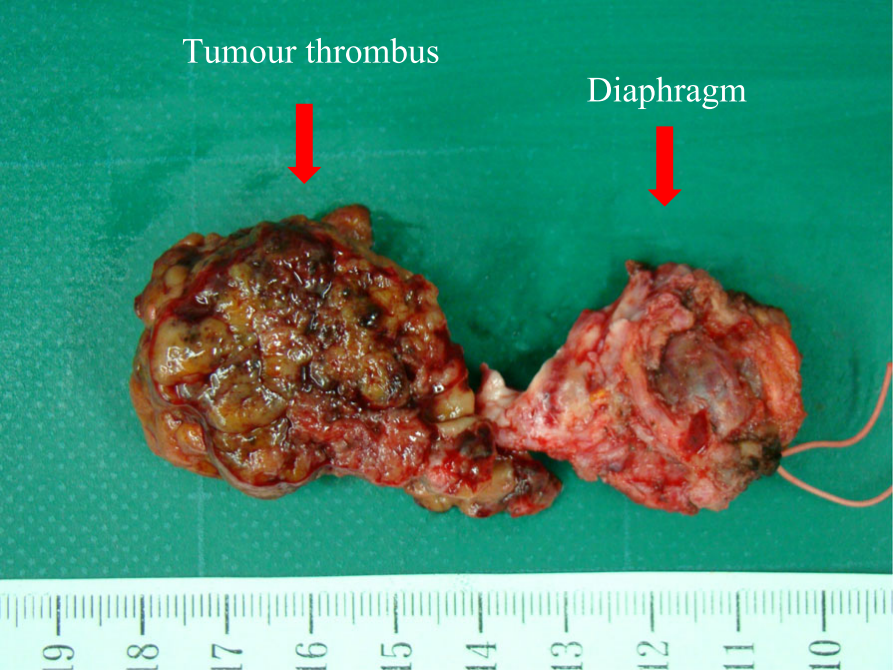
The resected specimen of case 1. It includes the tumor thrombus in the inferior vena cava (IVC), right atrium (RA), and diaphragm

**Fig. 6:**
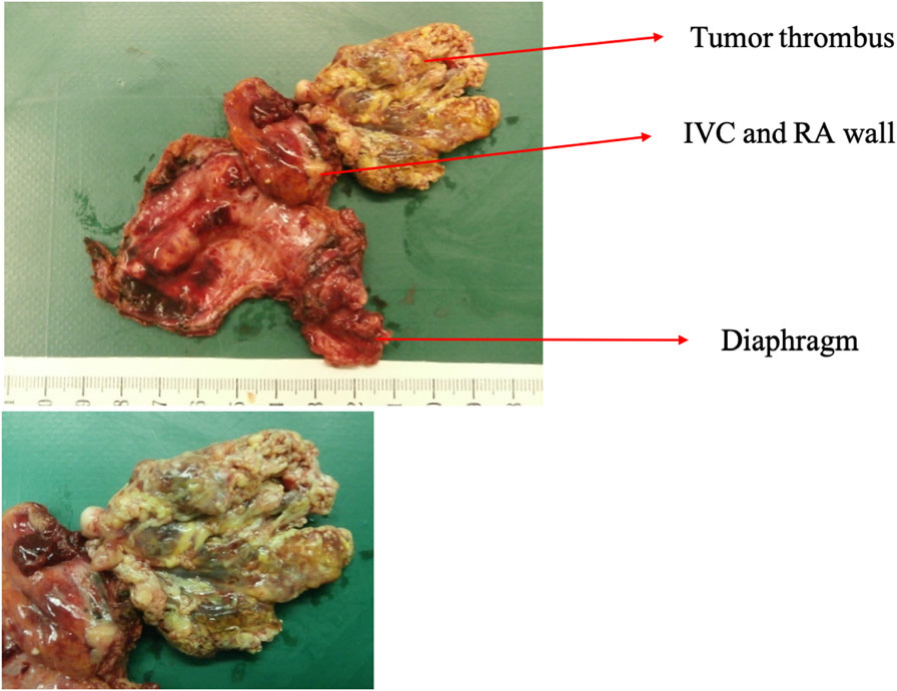
The resected specimen from case 3. It includes the tumor thrombus, inferior vena cava (IVC), right atrium (RA) wall, and diaphragm

The resected specimens of cases 1 and 3 are shown in Figs. 5 and 6, respectively (there were no available photographs for case 2). The tumors were not capsulized in all cases and were necrotic in cases 2 and 3. Furthermore, in all cases, tumor thrombi were recognized in the IVC and RA and inside the phrenic vein, where they had progressed extensively. Cases 1 and 3 had poorly differentiated HCCs, while case 2 had a moderately differentiated HCC. The microscopic surgical margins were positive in all three patients at the site of the resected diaphragm margins, whereas the tumor thrombi in the IVC and RA were totally resected.

### Postoperative course

Pleural effusions were postoperatively recognized in all three patients, though disappeared through a conservative approach. Patients 1, 2, and 3 were discharged on postoperative days 42, 14, and 43, respectively.

The postoperative levels of alfa-fetoprotein and protein induced by vitamin K absence-II tumor markers decreased compared to the preoperative values in all three patients, although they remained outside their normal ranges.

All patients were postoperatively administered sorafenib to prevent tumor progression, although the tumors progressed 3.8 to 20.2 months postoperatively in all cases. In case 1, recurrence was suspected at the parasternal site, although it had remained unchanged after the radiation therapy. In cases 2 and 3, the recurrences were detected in the lung and bone and the residual intrahepatic HCC, respectively, although there were no recurrences of the IVC thrombi. The overall survival was 98.8 months for case 1 (died of liver cirrhosis), 38.9 months for case 2 (died of acute heart failure), and 30.9 months for case 3 (died of HCC).

## Discussion

In this report, we presented the cases of three patients with recurrent HCCs and tumor thrombi in the IVC, RA, and phrenic vein. Tumor resections, including the tumor thrombus and diaphragm, were performed for these patients. Their prognosis was better than that of common Vv3 cases, in which the tumor thrombus progresses via the major hepatic veins.

In general, an HCC tumor thrombus of either the IVC or RA is uncommon and may result in intrapulmonary dissemination and/or pulmonary embolisms [2]. Additionally, a tumor thrombus of the RA may obstruct the orifice of the tricuspid valve, resulting in sudden cardiac arrest [3]. The median survival time (MST) of Vv3 (tumor thrombus in the IVC) has been shown to be less than 6 months, with no patients surviving beyond 2 years [7].

Treatment of Vv3 cases includes TACE [8], chemotherapy [9], stereotactic body radiotherapy [10], particle radiotherapy [11], and surgical resection. When compared with non-surgical treatments, tumor resections that include a tumor thrombus in the IVC or RA may improve the prognosis of patients with HCC by preventing sudden death [12]. Based on a nationwide Japanese survey, Kokudo et al. reported a better MST after surgical resections than after non-surgical treatments in Vv3 cases (1.48 vs. 0.84 years, *P* < 0.001) [13].

Tumor thrombi may rarely appear in the phrenic vein, and in such cases, the effect of surgical resections, including combined diaphragm resections, is unclear and has not previously been reported. The three cases presented here indicate that patients with a tumor thrombus in the IVC, RA, and phrenic vein may have a better prognosis when treated with surgical resections including the diaphragm. The improved prognosis may be related to the presumed slow growth of the tumor thrombus in these cases. According to the preoperative images of our cases, the primary masses and thrombi were hypovascular, appeared to be fed by the inferior phrenic artery, and the number of the arteries was very small. Additionally, the postoperative pathological findings showed that the tumors were necrotic, especially inside the masses and thrombi. These findings may support the assertion that recurrent HCCs grow slowly and are less vascular. Surgical resections may be effective for these slow-growing tumors, as they may permit a relatively longer survival, even if the tumor recurs. Moreover, in our cases, the tumor thrombi extended inside the phrenic vein continuously from the IVC or diaphragm. This finding also supports the effectiveness of surgical resections.

When considering the mechanisms of tumor progression in our patients, there are several speculations. Because the tumor mass was not recognized outside the IVC in case 1, it is possible that the tumor thrombus in the phrenic vein originated from a recurrence at the IVC wall. In previous reports, recurrent HCCs may have developed solitarily in the IVC [14] or in the heart [15]. It is also possible that the tumor thrombus had progressed from the intrahepatic or subphrenic tumor mass. Conversely, it might have extended to the diaphragm or subphrenic area.

The indication for surgical resections in these cases was difficult. In general, aggressive surgery for an HCC thrombus in the IVC is typically followed by early recurrence and subsequent difficulty in treatment of the recurrence site [16]. Other reports have indicated that surgical resection may be considered for the prevention of sudden death if distant metastasis and recurrence in the remnant liver are controlled [17]. In our cases, complete tumor resections could not be achieved, and the surgical margins were positive in all cases, especially in the combined resected diaphragm margins. However, we believe that surgical resection for the prevention of sudden death followed by intensive treatment, including chemotherapy and TACE, may improve patient prognosis. Additionally, the use of sorafenib as an adjuvant therapy might improve the prognosis in our cases, though the effectiveness of adjuvant therapies for hepatocellular resection has not been established.

There were possible postoperative complications related to the surgical procedure for these cases. Previous reports have observed pleural effusion, ascites [6], acute renal failure, and atrial fibrillation [16] as the postoperative complications. In the present study, pleural effusion was observed in all cases.

Finally, surgical resection of a tumor with a thrombus formation that has extended into the RA generally requires cardiopulmonary bypass [18]. Unfortunately, tumor resection under cardiopulmonary bypass is an invasive surgery with frequent postoperative complications. As indicated in this study, THVE is a useful procedure for tumor resections in the IVC [19–21]. If the systemic circulation is unstable or intestinal congestion has occurred, both passive veno-venous bypass from the infra-hepatic IVC and portal flow to the left axial vein can be performed simultaneously [22].

This study had some limitations. Only three cases were analyzed, making it difficult to conclude whether surgical resections are truly effective for recurrent HCCs with tumor thrombi in the IVC, RA, and phrenic vein. Therefore, further investigations will be needed to examine the usefulness of surgical resections in such cases.

## Conclusions

Surgical resections for recurrent HCCs with tumor thrombi in the IVC, RA, and phrenic vein may improve patient prognosis, although the indications should be considered carefully.

## Data Availability

All data generated or analyzed during this study are included in this published article.

## Abbreviations

HCC: Hepatocellular carcinoma
IVC: Inferior vena cava
MST: Median survival time
RA: Right atrium
TACE: Transarterial chemoembolization
THVE: Total hepatic vascular exclusion
